# A phase 2 trial of sunitinib in patients with progressive paraganglioma or pheochromocytoma: the SNIPP trial

**DOI:** 10.1038/s41416-019-0474-x

**Published:** 2019-05-20

**Authors:** Grainne M O’Kane, Shereen Ezzat, Anthony M. Joshua, Isabelle Bourdeau, Raya Leibowitz-Amit, Harold J. Olney, Monika Krzyzanowska, Dean Reuther, Soo Chin, Lisa Wang, Kelly Brooks, Aaron R. Hansen, Sylvia L. Asa, Jennifer J. Knox

**Affiliations:** 1 Department of Medical Oncology and Haematology Princess Margaret Cancer Centre, University Health Network, 610 University Avenue, Toronto Ontario M5G 2M9, Canada; 2 Endocrine Oncology Site Group, Princess Margaret Cancer Centre, University Health Network, 610 University Avenue, Toronto Ontario M5G 2M9, Canada; 3 Department of Medical Oncology, Kinghorn Cancer Centre, 370 Victoria St, Darlinghurst, NSW 2010 Australia; 4 Endocrinology Division, Department of Medicine, Centre de Recherche du Centre Hospitalier de l’Université de Montréal (CHUM), Montréal, Canada; 5 Department of Medical Oncology, Sheba Medical Center, Derech Sheba 2, Ramat-Gan, Israel; 6 Department of Medical Oncology, Tom Baker Cancer Centre, 1331 29 St NW, Calgary, Alberta T2N 4N2 Canada

**Keywords:** Adrenal tumours, Cancer genetics

## Abstract

**Background:**

Pheochromocytoma (PCC) and paraganglioma (PGL) are uncommon neoplasms with high morbidity in advanced stages. Effective systemic treatments are limited.

**Methods:**

A multisite phase 2 trial evaluated sunitinib in patients with progressive PCC/PGL. Patients received 50 mg orally for 4–6 weeks.

**Results:**

Between May 2009 and May 2016, 25 patients were enroled. The median age was 50 years and 56% were male. Three patients (12%) received prior chemotherapy and 16 (64%) prior surgery. The DCR was 83% (95% CI: 61–95%) and median PFS 13.4 (95% CI: 5.3–24.6) months. Of 23 evaluable patients, 3 (13%) with germline mutations (*SDHA, SDHB, RET*) achieved a PR. The patient with mutated *RET* and *MEN2A* remains on treatment after 64 cycles. The median time on treatment was 12.4 (1–88.0) months. Grade 3 or 4 toxicities were as expected and manageable; fatigue (16%) and thrombocytopenia (16%) were most common. One patient with grade 3 hypertension and 2 with grade 3 cardiac events discontinued treatment.

**Conclusion:**

Although the primary endpoint of disease control was met, the overall response rate of sunitinib was low in unselected patients with progressive PCC/PGL. Patients with germline variants in *RET* or in the subunits of *SDH* may derive greatest benefit.

## Background

Pheochromocytomas (PCCs) and extra-adrenal paragangliomas (PGL) are uncommon neuroendocrine neoplasms arising from the adrenal medulla and sympathetic/parasympathetic paraganglia respectively. Local treatments including surgical resection, radiation and radiolabelled agents are important therapeutic options when possible. Up to 20% of PCC/PGL can present with bulky inoperable disease, progressive symptoms or the presence of metastases, indicating high morbidity and a 5-year survival of less than 50%.^1^

In over 30% of cases of PCC/PGL, a predisposing germline mutation can be identified, the most common are inactivating mutations in subunits of the succinate dehydrogenenase (SDH) complex predominantly *SDHB* and *SDHD*.^2,3^ Other hereditary susceptibility genes include *RET* and *von Hippel-Lindau* (*VHL*) as part of the syndromes of multiple endocrine neoplasia 2 (MEN2) and VHL syndrome respectively; the tumour suppressor gene *neurofibromatosis 1* (*NF1*),^4^ *MYC associated factor X* (*MAX*) and *transmembrane protein 127* (*TMEM127*) and a number of additional genes.^3^ Recent molecular characterisation and mRNA expression profile clustering have identified germline *VHL* and *SDH* subunit mutations specific to a pseudohypoxia subgroup of PCC/PGL, or cluster 1 as previously published.^5^ Both genes are important in the regulation of hypoxia inducible factor (HIF) and angiogenesis.^6,7^ Subsets of sporadic PCC/PGL are also thought to exhibit high expression of angiogenic factors.^8^ Accordingly, case reports and small series have suggested a role for anti-angiogenic agents in treating this group of endocrine tumours.^9–11^

Systemic options for advanced PCC/PGL are limited. No prospective trials have been published to guide treatment decisions. Variations of the cytotoxic regimen of cyclophosphamide, vincristine and dacarbazine (CVD), and temozolomide regimens have been reported in small retrospective series and case studies, with variable response rates and limited quality data.^12–17^ In addition chemotherapy-related toxicities are common, including myelosuppression, neuropathy and gastrointestinal toxicity.^12^ Better treatment options are urgently needed. Sunitinib is an oral tyrosine kinase inhibitor targeting vascular endothelial factor receptor 1 and 2, PDGF-B receptor, RET and other tyrosine kinases including FGFR that is overexpressed in PCC/PGL.^18^ We conducted a multicentre open label single arm phase 2 study of sunitinib in patients with locally advanced unresectable or metastatic PCC/PGL who had demonstrated progression or who had disease-related symptoms.

## Methods

### Patients

Patients included in the study were ≥18 years of age, Eastern Cooperative Oncology Group (ECOG) performance status 0–2, with a histologically or cytologically confirmed diagnosis of PCC/PGL that was considered non-resectable or metastatic. To be enroled patients needed measurable disease and demonstrated radiological progression by RECIST1.1 criteria and/or biochemical progression. The protocol was later amended to allow newly presenting patients with tumour-related symptoms without radiological progression. Radiological progression was defined by the presence of new lesions or a 20% increase in the sum of the longest diameter of target lesions, comparing two computed-tomography (CT) scans performed within 13 months of each other. This time frame was included given that this patient population is scanned infrequently. These pre-treatment measurements were recorded to ascertain rate of growth over time, prior to sunitinib and comparing to on-treatment measurements. Biochemical progression was defined as a change ≥50% in urinary catecholamines and/or measured urinary metanephrines over 5–7 months. Adequate organ function was required, and patients were excluded for prior anti-angiogenic treatment, uncontrolled hypothyroidism, HIV infection, venous thromboembolism within 3 months or any major vascular event. Notably hypertension must have been controlled prior to enrolment (systolic ≤ 150 mmHg and/or diastolic ≤ 90 mmHg). CYP3A4 inhibitors and inducers were prohibited 7 and 12 days respectively prior to study commencement and throughout the trial. Patients with QTc prolongation (≥500 ms) or those receiving pro-arrhythmics were also excluded. Germline testing was not a study requirement however in those patients who did undergo germline evaluation, testing was performed as per institutional guidelines.

### Study design

#### Procedures

In this single-arm multi-institutional phase 2 study, all patients received starting dose of sunitinib 50 mg orally, daily for 4 weeks, followed by 2 weeks observation; each cycle was 6 weeks in duration and was maintained even when doses were missed during the 4-week treatment period. Within 7 days of enrolment and prior to each cycle, all patients were required to have had a physical examination, blood chemistry including TSH and T4, and urinary analysis for protein excretion. Every second cycle, analysis of 24-h urinary collection for metanephrines and catecholamines, serum chromogranin A, together with a CT chest abdomen and pelvis were performed.

At week 2 and week 4 of treatment, patients had blood pressure and heart rate checks. Guidelines for the management of hypertension including the appropriate antihypertensives to be prescribed and required dose reductions were available within the protocol. In general blood pressure was to be maintained at less than 150/90 mmHg. Dose modifications were made for other grade 3 or 4 adverse events. Patients discontinued treatment for unacceptable toxicity, disease progression, investigator discretion or consent withdrawal.

### Outcomes and statistical analyses

The primary endpoint of this study was disease control rate (DCR) defined as a partial response (PR), complete response (CR), or stable disease maintained for ≥12 weeks from the initiation of treatment. Tumour response to sunitinib was assessed as per RECIST 1.1 using CT-imaging. Clinically apparent lesions needed to be >10 mm by calliper measurement to be included as targets. Secondary endpoints included biochemical response as defined by >20% reduction in one of the following; 24-h urinary catecholamines (norepinephrine, epinephrine, dopamine) or total metanephrines, which was sustained for ≥12 weeks; progression free survival (PFS), overall survival (OS), overall response rate (ORR) and symptom control. Adverse events were assessed according to the revised NCI Common Terminology Criteria for Adverse Events (CTCAE), version 3.0 during treatment and through 28 days post discontinuation of sunitinib.

Data from all participating study sites were combined for the analyses. Summary data are reported for baseline demographics, efficacy and safety data. Categorical data are presented as frequencies and percentages and continuous data as the median and range unless stated otherwise. A multi-stage design as described by Fleming^19^ was employed, assuming an unacceptable response rate (defined by DCR) of 5% and a promising rate of 20% with type I and type II errors of 10% each. 14 response evaluable patients were entered in the first stage to accrue to the second stage if ≥1 response as defined by DCR was seen. The primary endpoint was met if ≥4 responses (as defined by the DCR) were found after a total enrolment of 28 patients. The percentage change in volume over time was plotted using a spider plot to assess antitumor activity with pre-treatment measurements also included. The Kaplan Meier method was used to assess PFS and OS. PFS was defined as the time from registration to progression or death from any cause and OS, the time from registration to death from any cause. Data lock occurred on the 31 January 2018.

### Study oversight

The study was approved by the appropriate ethics committee or institutional review board at each study site. The study was conducted in accordance with Guidelines for Good Clinical Practice. All patients provided written informed prior to enrolment.

## Results

### Patient characteristics

In total, 25 of the planned 28 patients were enroled between May 2009 and May 2016 from three centres within Canada and one in the Netherlands. The study was closed on the 18th January 2017 due to slow accrual, demographics and clinical characteristics are shown in Table 1. Fourteen patients had PCC (56%) and 11 PGL (44%). Twenty-three (92%) had metastatic disease and 2 patients had locally advanced disease. The majority (*n* = 20, 80%) of patients were enroled based on radiologic and/or biochemical progression. Five patients (20%) presenting with a recent diagnosis of advanced PCC/PGL were enroled based on rapid symptomatic progression. One patient (SNP-010) had evidence of progressive metastatic disease to bone only together with biochemical progression and although did not have measurable disease by RECIST at baseline, was allowed to enrol given burden of pain related symptoms.

**Table 1:** Baseline characteristics of patients enroled

Characteristic	Patients *N* (%)
Median age, years (range)	50 (17–79)
Sex	
Male	14 (56)
Female	11 (44)
ECOG	
0	11 (44)
1	10 (40)
2	4 (16)
Histology	
Paraganglionoma	11 (44)
Pheochromocytoma	14 (56)
Prior systemic therapy	
Systemic adjuvant	1 (4)
Systemic advanced	3 (12)
None	21 (84)
Prior local therapy	
Radiation only	4 (16)
Surgery only	11 (44)
Surgery and radiation	5 (20)
None	5 (20)
Stage	
Metastatic	23 (92)
Locally advanced	2 (2)
Sites of metastases	
Lymph nodes	11 (44)
Lung	12 (48)
Bone	12 (48)
Liver	14 (56)
Total baseline urinary metanephrines elevated	
Yes	15 (60)
No	6 (24)
Missing	4 (16)
Reason for enrolment	
Radiologic and/or biochemical PD	20 (80)
Symptomatic PD	5 (20)

Genetic testing results were available in 60% (15/25) of patients. Of these 15 patients, 9 (60%) had germline mutations identified, including 5 *SDHB* mutations, 1 *SDHA* mutation, 1 *SDHC* mutation, 1 *RET* mutation in a patient with MEN2A and 1 *MAX* mutation. The median time from first diagnosis to enrolment was 33.6 months (3– >132). Surgical resection of primary or metastatic sites had been previously performed in 16 (64%) patients. Three patients received prior chemotherapy for advanced disease, 1 patient received cisplatin/vinorelbine, 1 patient CVD and 1 patient had received two prior regimens including carboplatin/etoposide, and temozolomide/capecitabine.

### Treatment outcomes

**Fig. 1:**
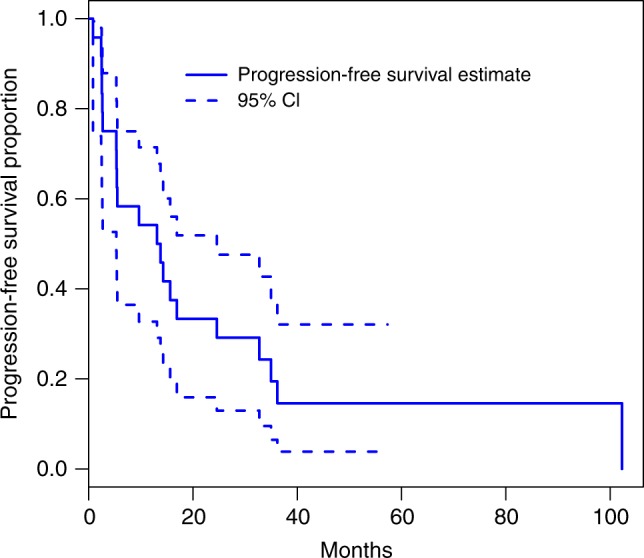
Kaplan Meier plot of progression-free survival through to 31 January 2018

**Fig. 2:**
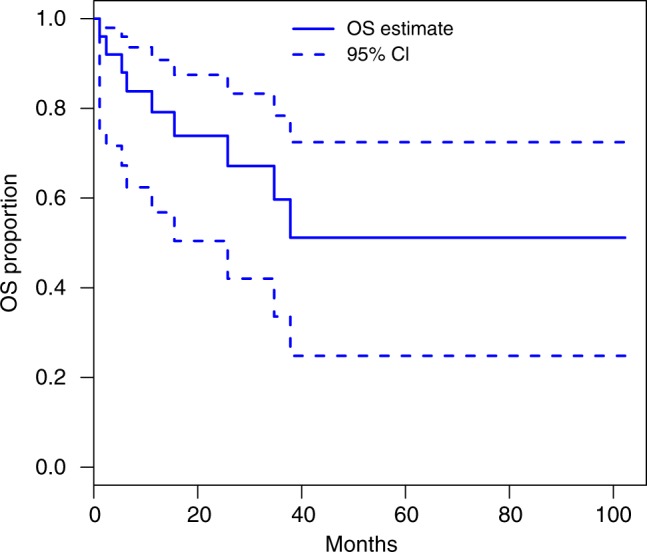
Kaplan Meier plot of overall survival through to 31 January 2018

The median duration on treatment was 12.4 (1–88.0) months at data cut-off (Table 2). The median dose intensity was 40 mg (25 mg−50 mg) with 14 patients (56%) requiring dose reductions. Twenty-three patients were evaluable for response. Three patients (13%) achieved a partial response and 16 (70%) had stable disease ≥12 weeks as best response. The objective response rate (RECIST) was 13% and the DCR was therefore 83% (95% CI: 56–93%), meeting our primary endpoint. At 24 weeks 61% had evidence of disease control. The median PFS was 13.4 (95% CI: 5.3–24.6) months; 21 of 25 patients had a PFS event (1 death and 20 patients had disease progression, four patients are censored including two who remain on study and two who were lost to follow-up, Fig. 1). The median overall survival has not yet been reached; 9 (36%) patients have died (Fig. 2).

**Table 2:** Response to treatment (*N* = 25; 23 evaluable for response data)^a^

Response	
Best response	
CR	0 (0)
PR	3 (13)
SD > 12 wks.	16 (70)
PD	4 (17)
Unknown	2
Disease control rate	
(CR, PR, SD > 12 weeks)	19 (83%)
(95% CI)	(61–95%)
Overall response rate	
(CR+PR)	3 (13%)
(95% CI)	(0.03–0.34)
PFS events	
Progression	20 (80)
Death	1 (4)
Censored	4 (16)
(2 lost to follow-up 2 remain on study)	
Median PFS, mths (95% CI)	13.4 (5.3–24.6)
Median time on treatment, mths (range)	12.4 (1.0–88.0)

^a^SNP-10 did not have measureable disease at baseline but is included in the 23

**Fig. 3:**
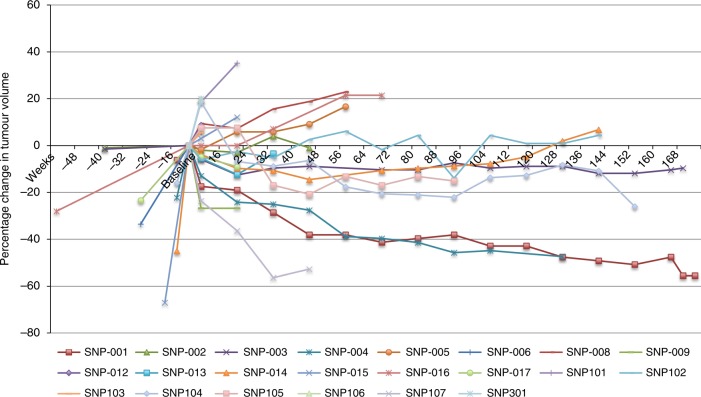
Spider plot showing responses to treatment over time and pre-trial tumour growth

**Fig. 4:**
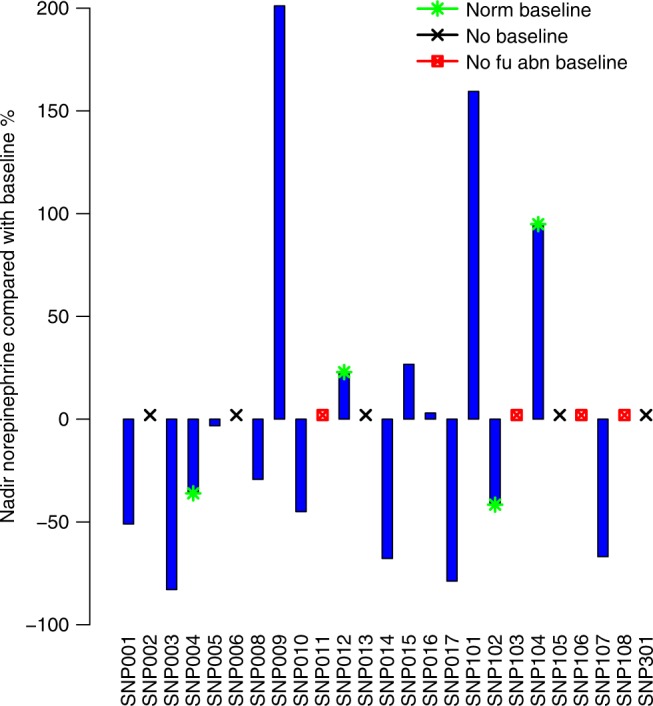
Waterfall plot showing % change in 24-h urinary metanephrine results. Those with no baseline values or follow-up results are indicated. The median (range) cycle to nadir for patients with abnormal baseline results with at least 1 follow-up result (*n* = 12) 3 (2, 45)

In the 3 patients achieving a PR, all three were found to have germline mutations. A patient with PCC who had a *RET* mutation (SNP-001) had a total reduction in tumour volume of 64% as best response and remains on treatment after 64 cycles (7 years) (Fig. 3). This patient also had significant biochemical responses (Fig. 4 and Supplementary Fig. 1–3). The second patient (SNP-004) with PCC who had a germline *SDHB* mutation had a 47% reduction in tumour volume as best response after 23 cycles but stopped treatment due to progression in non-target lesions together with evidence of clinical progression. Both of these patients reported improvements in pain with SNP-001 reporting a complete resolution in pain related to disease burden. The third patient (SNP-107, PGL) with a germline *SDHA* mutation who after 7 cycles had a 56% reduction in target tumour volume, subsequently developed new lesions after 10 cycles of sunitinib.

Sixteen patients achieved stable disease ≥ 12 weeks as best response by RECIST 1.1 criteria. Of these patients 6 had reductions of tumour volume greater than 10% (range: 12–27%). Three of these patients had germline mutations in *SDH*. In the 6 remaining patients with identified germline mutations in predisposition genes, 4 patients (3 *SDHB* and 1 *SDHC*) had prolonged stability by RECIST 1.1 lasting beyond 16 cycles with one patient remaining on treatment after 33 cycles (SNP-010). This patient who did not have measurable disease at baseline reported significant reductions in pain. One additional patient with an *SDHB* mutation had a short period of disease stability, stopping treatment for progression after four cycles. One further patient with a *MAX* mutation progressed on treatment. Disease stability was seen in ten further patients.

### Biochemical and symptom changes

Timed urinary catecholamine collection over 24 h included measurements of norepinephrine, epinephrine, dopamine and total metanephrines. Three patients had normal results and 6 had no follow-up results recorded; of 16 evaluable. 11 had evidence of a biochemical response. Fractionated urinary metanephrines were captured in some patients but were not measured routinely at opening of the trial. Reference ranges varied between institutions. Elevated levels of total 24-h urinary metanephrines were evident in 15 of 21 patients with available baseline values (71%). Of these 15, 12 had subsequent values recorded, of which seven (58%) (3 PGL, 4 PCC) had a greater than 20% decline in values more lasting more than 12 weeks (Fig. 4). One of these 7 patient (SNP-001) also had a RECIST response. The two other patients with partial responses had normal baseline metanephrines. The three patients without subsequent values had received only 1–2 cycles of treatment with evidence of clinical or radiologic progression.

Baseline levels of norepinephrine were available in 20 patients (80%); of these 16 patients had elevated levels. Twelve of 16 patients (75%) had follow-up results, of these 7 patients had a greater than 20% biochemical reduction. The median cycle to best response was achieved at cycle 3 (range: 3–30, Supplementary Fig. 1). Epinephrine baseline results were available in 19 (76%) patients of which 6 had abnormal values. One patient had no follow-up results and 4 of 5 patients had evidence of a biochemical response, the median cycle to response was 4 (Supplementary Fig. 2). Dopamine urinary results were abnormal in 8 of 20 patients with available baseline results. Three of 5 patients with follow-up measurements had a biochemical response (Supplementary Fig. 3). Follow-up values were predominantly not available because of study cessation or progressive clinical decline.

In the five patients enroled based on symptoms only, the median number of cycles received was 4 (2–10). Three of these patients had an ECOG of 2 at diagnosis without improvement on study; two reported reductions in pain scores.

### Toxicity

Reported AEs are shown in Table 3. The AEs were as expected for sunitinib and manageable. The most common grade 1–2 AEs were fatigue (68%), nausea and/or vomiting (68%) and palmar-plantar dyserthrodysesthesia (64%). Grade 3 events occurred in 14 (56%) patients and grade 4 in 3 patients (12%). Five (20%) patients discontinued sunitinib because of adverse events, including 1 patient with grade 2 nausea/vomiting, 1 patient with grade 2 elevation in creatinine and 1 patient with grade 3 hypertension. Two cardiac events occurred leading to discontinuation; 1 patient developed a restrictive cardiomyopathy related to study drug and one patient also had a myocardial infarction following episodic hypertensive episodes. There were no grade 5 events.

**Table 3:** Adverse events suspected to be related to sunitinib

Adverse event	*N* = %	
	All grade	Grade 3 or grade 4
Fatigue	17 (68)	4 (16)
Nausea/Vomiting	16 (64)	2 (8)
PPES	16 (64)	2 (8)
Diarrhoea	11 (44)	1 (4)
Hypertension	11 (44)	2 (8)
Mucositis	10 (40)	1 (4)
Dysguesia	7 (28)	0 (0)
Anorexia	5 (20)	0 (0)
Thrombocytopenia	6 (24)	4 (16)
AST/ALT increased	3 (12)	1 (4)
Anaemia	3 (12)	2 (8)
Hypothyroidism	3 (12)	0 (0)
Neutropenia	3 (12)	1 (4)
Elevated creatinine	3 (12)	0 (0)
Hyponatraemia	2 (8)	2 (8)
Cardiac ischaemia^a^	1 (4)	1 (4)
Cardiomyopathy	1 (4)	1 (4)

All patients were counted once at the highest grade for each preferred term Adverse events were graded according to National Cancer Institute Common Terminology Criteria for Adverse Events (version X)

*PPES* palmar-plantar erythrodysesthesia syndrome

^a^The cardiac ischaemic event occurred following episodic hypertensive episodes

All grade 3/4 events are reported, only grade 1/2 occurring in more than 2 patients are documented

## Discussion

Pheochromocytoma (PCC) and paraganglionoma (PGL) are rare neuroendocrine neoplasms with limited systemic options in the setting of progressive inoperable or metastatic disease. Comprehensive molecular profiling together with increased germline detection has allowed for a more complete understanding of this heterogeneous group of tumours, providing potential biomarker-derived subgroups for drug targets.^5^

This phase 2 trial met its primary endpoint; however objective response rates has of sunitinib in an unselected group of patients with progressive and/or symptomatic disease were low. Durable responses, however were seen in one patient with a *RET* mutation and two with *SDH* mutations, suggesting potential biomarkers of response to this agent. This study represents the largest prospective analysis of a multi tyrosine kinase inhibitor in advanced PCC/PGL. The disease control rate was 83% and median PFS 13.4 months at data-cut-off. In addition, biochemical responses were evident in 11/16 (69%) evaluable patients.

As demonstrated in the spider plot, there is a change in the growth kinetics of a number of these cases, suggesting a durable disease control benefit from sunitinib rather than selection of patients with indolent disease. SNP-004 and SNP-104 had evidence of 22 and 16% growth in tumour volume approximately 12 weeks prior to study entry and subsequently had best tumour responses of −47 and −26% respectively. SNP-001 had growth of 6% in the prior 12 weeks and has had tumour shrinkage > 55%, remaining on treatment. The subset (*n* = 5) enroled for tumour-related symptoms appeared to have the shortest exposure to sunitinib with fairly early discontinuation due to functional decline and progressive disease. Patients were not required to undergo genetic testing as part of this study however of the three patients with a partial response, germline mutations in *SDHB*, *SDHA* and *RET* were evident. In the patient with MEN2A syndrome, the response is ongoing for more than 7 years. Prolonged stability was also present in four other patients with germline *SDH* mutations. An additional 10 patients in whom genetic testing results were not available or negative had stable disease as best response.

The results of our prospective trial are corroborated in other series of multi-targeted tyrosine kinase inhibitors.^11,20^ In the combined retrospective analysis from MD Anderson and Institute Gustavvy-Roussy, 3 patients (18%) of 17 reported developed a partial response and an additional 5 patients had disease stability resulting in a DCR of 47%. Of the 8 patients achieving disease control, 6 had germline inactivating mutations in *SDHB* or *VHL* genes.^11^ In this study the PFS was 4.1 months, attributed to early cessation due to side effects. Evaluation of sunitinib in our clinical trial, where blood pressure was rigorously controlled has allowed for a more accurate determination of efficacy. Although grade 3 adverse events were present in over 50%, the sample size in this study is small and similar percentages have been reported.^21^

The *SDH* genes which include SDHA, SDHB, SDHC, SDHD encode the subunits of the succinate dehydrogenase complex (SDH) together with the assembly factor SDHAF2. Recent molecular characterisation of PCC/PGL identifies four major subgroups by unsupervised clustering of mRNA expression profiles. Germline SDH and VHL mutations are represented in the pseudohypoxia group or cluster 1 supporting prior studies.^5,22–24^ Disruption of the SDH or mitochondrial II complex results in the accumulation of succinate which in turn inhibits HIF-alpha prolyl hydroxylases activating HIF-1 alpha which in turn autoregulates the cycle suppressing SDHB levels.^6,24^ Stabilisation and activation of the transcription factor HIF-1 alpha regulates the hypoxic response and as a result upregulates multiple factors involved in angiogenesis, including VEGF and PDGF.^25^ Similarly in the presence of VHL mutations, HIF-1 alpha and HIF 2- alpha become constitutively active given the failure of proteasomal degradation and polyubiquitination.^26^ The response seen in our study in the pseudohypoxia group which is frequently enriched in angiogenic pathway activation, is akin to that seen in renal cell cancer where sunitinib has been a standard of care for many years.

Relevant to this trial, mutations in *RET* are categorised within a kinase signalling subtype also known as cluster 2.^22–24^ Notably the best responder in this study was a patient with a mutation in RET and MEN2A. Sunitinib is tyrosine kinase inhibitor that also targets RET.^27^ This suggests that sunitinib in addition to targeting pseudohypoxia subgroups may have additional activity in patients with *RET* mutations.

Algorithms for the treatment of advanced PCC/PGL are limited by a lack of prospective studies. Chemotherapy (CVD) in this population has been reported in a retrospective study to provide response rates of 55% however survival at 5 years was less than 50%.^12^ In the largest institutional experience with chemotherapy to date, 33% of patients had a response defined as tumour shrinkage or normalised blood pressure. Of the 54 patients in this study 24% had tumour shrinkage and 51% were alive at 5-years.^15^ Similarly in a retrospective study of temozolomide in metastatic PGL/PCC response rates were 33% and median PFS 13.4 months similar to the PFS in this study. These survival outcomes are similar to that reported in our study.

The response rate to ^131^I-MIBG in patients with metastatic PCC/PGL demonstrating ^123^ I-MIBG avidity was 22% with an estimated 5-year survival of 64%.^28^ In that study two patients died from myelodysplastic syndrome and one as a result of ARDs, most patients received 1–2 infusions only.^28^ Notably SDHB- associated PCC/PGL were more likely to respond to ^131^I-MIBG. Although response rates to sunitinib in our study were less than those described above, the median number of cycles in patients with a germline mutation was 22 (1–64), more than 2.5 years with one patient on treatment more than 7 years with a significant reduction in tumour volume.

Definitive prognostic variables in PCC/PGL are limited and the true survival when progressive and/or metastatic disease is present has been difficult to determine due to study biases and the heterogeneity in definitions of metastatic disease.^29^ Despite this, tumour progression is the most frequent cause of death in patients with advanced PCC/PGL underscoring the need for systemic agents.^30^

This study has several limitations. For a variety of reasons, a proportion of patients did not have genetic testing and given the small numbers, this trial is not able to define prognostic or predictive biomarkers. Furthermore, genetic panel testing may have varied between institution and during the study period. Biochemical response assessment was limited because plasma metanephrine analysis was not performed throughout the study and only total but not fractionated urinary metanephrines were available.^31^ Given the multisite nature of the study, a number of patients did not have either baseline or follow-up samples which makes biochemical interpretation less than complete. Furthermore, plasma metanephrines/normetanephrine or their fractionated urinary counterparts are now standard practice in this population,^31^ but this was not part of the study protocol in 2009. Hypertension in this trial must have been adequately controlled prior to study entry, and as a result only two patients experienced grade 3/4 hypertension. Patient-reported outcomes were not integrated into the study which was initiated in May 2009. It should however be noted that 3 patients reported significant reductions in pain scores. Furthermore, the improvement in symptoms and health-related quality of life could have been under-reported. Although our study, like others, did not fully accrue,^20^ this trial represents the largest prospective study of a targeted approach in patients with progressive PCC/PGL. The low response rates suggest the need for a greater understanding of the heterogeneity of the disease and the molecular drivers. Dual inhibition or multi targeted agents may be required to overcome drug resistance and improve efficacy.^32^

In summary, sunitinib in this prospective trial had modest activity as a single agent in an unselected population and could be considered a systemic option. The FIRSTMAPPP trial which is randomising sunitinib at a dose of 37.5 mg to placebo will help establish the role of sunitinib in this setting. While those with germline mutations in the SDH subunit genes and pseudohypoxia signatures may derive great benefit, we also show that *RET* mutation may be a biomarker for response to this agent that can target other pathways implicated in the kinase signalling subtype. Larger collaborative efforts will be needed in the future to conduct prospective biomarker-driven trials in this rare, molecularly heterogeneous group of neuroendocrine neoplasms.

## Supplementary information

Supplemetary Figures 1-3

## Data Availability

This study is available withinthis publication and in the supplementary material. Further patient data can be requested from the corresponding author.
