# Supplementary material for: A phase 2 trial of sunitinib in patients with progressive paraganglioma or pheochromocytoma: the SNIPP trial
Source: Br J Cancer. 2019 May 20;120(12):1113–9. doi: 10.1038/s41416-019-0474-x (PMC6738062; doi:10.1038/s41416-019-0474-x)
Supplement: Supplementary file 1 — Supplemetary Figures 1-3 [file 41416_2019_474_MOESM1_ESM.docx]

Supplementary Figures 1-3


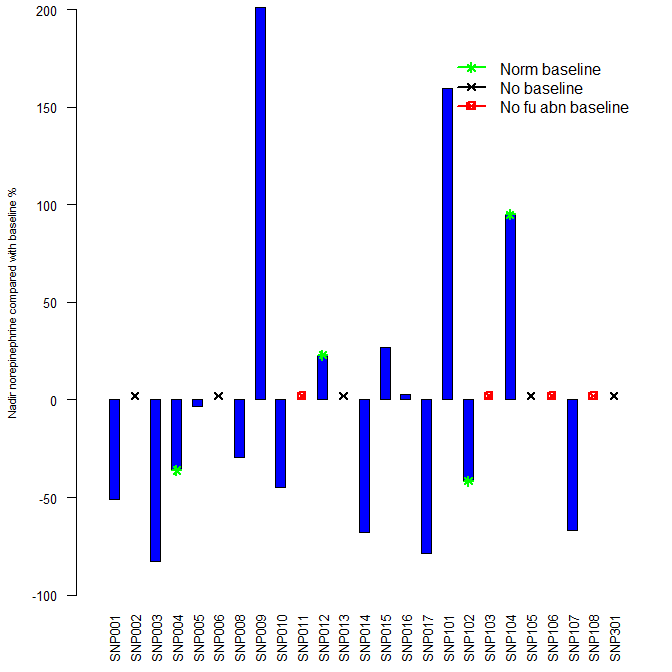


Figure S1: Waterfall plot showing % change in 24 hour urinary norepinephrine results. Those with no baseline values or follow-up results are indicated. The median (range) cycle to nadir for patients with abnormal baseline results with at least 1 follow-up result (n=12) was 5 (3, 30).


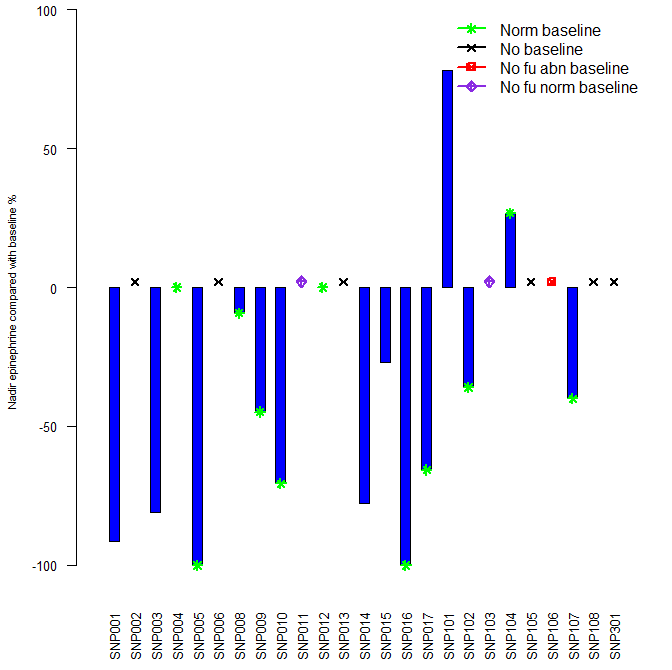


Figure S2: Waterfall plot showing % change in 24 hour urinary epinephrine results. Those with no baseline values or follow-up results are indicated. The median (range) cycle to nadir for patients with abnormal baseline results with at least 1 follow-up result (n=5) was 4 (3, 31).


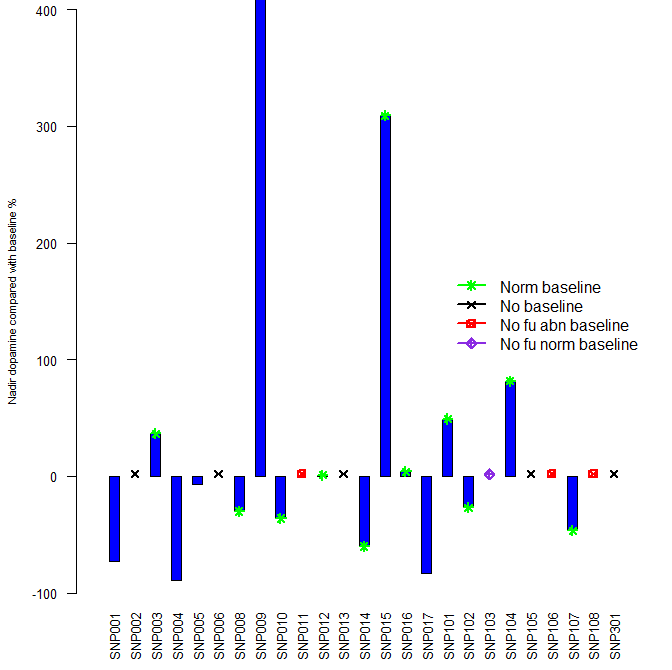


Figure S3: Waterfall plot showing % change in 24 hour urinary dopamine results. Those with no baseline values or follow-up results are indicated. The median (range) cycle to nadir for patients with abnormal baseline results with at least 1 follow-up result (n=5) was 4.5 (3, 30).
